# The Management of Gas-Filled Eyes in the Emergency Department

**DOI:** 10.1155/2014/347868

**Published:** 2014-11-26

**Authors:** Lik Thai Lim, Elliott Y. Ah-kee, Beve P. House, Jonathan D. Walker

**Affiliations:** ^1^Tennent Institute of Ophthalmology, Gartnavel General Hospital, 1053 Great Western Road, Glasgow G12 0YN, UK; ^2^Monklands Hospital, Monkscourt Avenue, Airdrie, North Lanarkshire ML6 0JS, UK; ^3^Lutheran Hospital, Fort Wayne, IN 46804, USA; ^4^Indiana University School of Medicine, Fort Wayne, IN 46202, USA

## Abstract

*Background*. Intraocular gas bubbles are commonly used in retinal surgery. There are specific management guidelines that need to be followed to ensure surgical success, and there are also unique ophthalmic and systemic complications that can occur in such patients. *Objective*. To educate emergency department personnel about important issues in the management of patients who have a gas-filled eye following retinal surgery. *Case Report*. A patient with a gas-filled eye developed several complications including pain, severe vision loss, high-grade atrioventricular (AV) block, and pneumocephalus. *Conclusion*. Awareness of potential problems that may arise in patients with gas-filled eyes who present to the emergency department may help minimize morbidity for such patients.

## 1. Introduction 

It is important to determine if a gas bubble has been placed in the eye of a patient with recent retinal surgery, because such patients may have special requirements and problems that can alter care provided in the emergency department.

## 2. Case Report

An 84-year-old white female underwent surgical repair of a macular hole in her left eye. The surgery consisted of a vitrectomy followed by the instillation of a mixture of C3F8 gas and sterile air into the vitreous cavity. After such a procedure, the patient is asked to maintain a face-down position for several days, which facilitates hole closure and improves vision. The gas bubble is then gradually absorbed over a period of weeks and the eye returns to a fluid-filled state. The patient's past medical history was notable for long-standing coronary artery disease, hypertension, diabetes mellitus, and a past surgical history of right carotid endarterectomy. The surgery was uneventful, but on postoperative day one the pressure in the eye was elevated. The surgeon performed an anterior chamber paracentesis to normalize the pressure. That evening the patient presented to the ED with a headache and symptomatic bradycardia with a heart rate of 25. She was noted to have high-grade AV block with AV dissociation and intermittent idioventricular escape. She underwent implantation of a dual-chamber permanent pacemaker, which corrected her dysrhythmia. During this period of time she was unable to maintain the recommended position for the retinal surgery.

**Figure 1:**
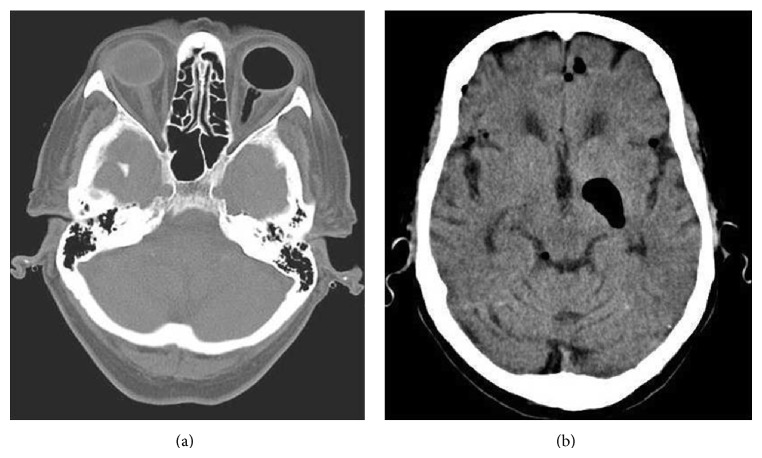
CT scan showing gas in the left eye and optic nerve (a) and intracranial air adjacent to the basal ganglia (b).

Her retinal surgeon was not informed of these events and she was ultimately seen again on postoperative day five. On that visit her anterior chamber was flat. (The iris and her intraocular lens had been pushed forward against the back of the cornea. This was an indication that the gas bubble had significantly expanded or she had been positioning improperly, with the bubble pushing up against the front of the eye rather than back toward the macular hole.) The pressure in the eye was markedly elevated to 88 (normal being up to 21). The ophthalmologist lowered the pressure to the normal range by withdrawing gas from the vitreous cavity. Later that day the patient developed acute mental status changes and again presented to the ED. A computed tomography (CT) scan demonstrated intracranial air in addition to the gas bubble in the eye (Figure 1). She was referred to a tertiary care center where it was opined that the intraocular gas bubble had expanded and tracked along the optic nerve, eventually entering the brain. This was likely due to the use of an expansile gas concentration during the surgery. The intracranial air gradually resolved, as did her mental status changes, but the involved eye lost all vision due to high pressure maintained over an extended period of time.

A case of pneumocephalus following macula hole repair has previously been published in the ophthalmology literature because it was opined that an incorrect gas concentration was instilled in the eye, resulting in marked expansion of the gas bubble [1]. Our case is presented here because gas bubbles are frequently used in retinal surgery, and there are specific management issues that apply to such patients in the ED. To our knowledge there is no discussion of such cases in the emergency medicine literature.

## 3. Discussion

**Figure 2:**
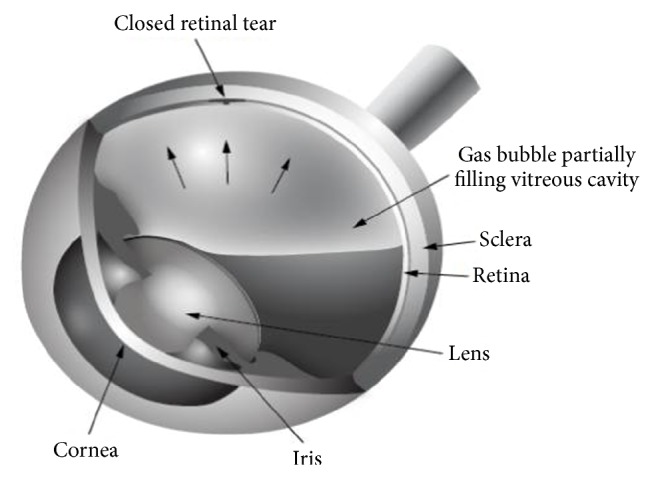
Drawing showing the appearance of an intraocular gas bubble. The surface tension of the air bubble helps to seal a retinal tear. This allows for the use of laser or cryotherapy to scar down the retina surrounding the hole, so the retina remains attached after the bubble is absorbed.

There are two main indications for inserting gas into the vitreous cavity: treatment of a retinal detachment and repair of a macular hole. The gas used is either SF6 or C3F8, mixed with sterile air to create a nonexpansile concentration (although sometimes a smaller amount of pure gas is injected in the eye in a procedure known as pneumatic retinopexy). The intraocular bubble can last up to 2 months depending on the amount placed in the eye and the type of gas used. While in the eye, the bubble is able to act as a tamponade to the retinal defect being treated (Figure 2). In the context of a retinal detachment, cryotherapy or laser is used to create fibrosis that permanently holds the retina in position—a process that takes between five and fourteen days. In the case of macular hole surgery, no cryotherapy or laser is used. The gas bubble simply creates an air-fluid interface that facilitates hole closure. In both cases, the patient can be asked to maintain some degree of face-down positioning. Sometimes the patient is required to position on one side or the other depending on the location of the pathology. However, there is currently no strong evidence to suggest that positioning is critical in macular hole surgery or even vitrectomy for retinal detachment. Positioning is more important in pneumatic retinopexy, which has been widely abandoned in Europe due to its low success rate [2]. Moreover, in cases of submacular hemorrhages, the patient should be asked to look straightforward for optimal pneumatic displacement of the hemorrhage and face-down positioning should be avoided [3].

In cases where appropriate positioning of the patient is required, this should be taken into account when treating other medical problems to avoid failure of surgery. The potential for complications is exacerbated if patients spend much time lying with their face up. In this case the bubble will push against the front of the eye rather than back against the retina. This can result in cataract formation if the patient has not had cataract surgery. However this depends on the duration of contact between the lens and the gas, which is longer in the face-up position. In pneumatic retinopexy, there is almost no cataract formation whereas, in macular hole surgery, nearly all phakic eyes will develop significant cataract within 6 to 12 months. Moreover, the pressure of the bubble against the lens and iris can push those structures forward resulting in secondary angle closure glaucoma. This in turn can cause irreversible vision loss from elevated intraocular pressure if it is not identified and treated quickly [4, 5].

The mechanism for air, silicone oil, or perfluorooctane migration from the intraocular space into the optic nerve and brain is not well understood and is very rare. This may occur in patients with optic nerve abnormalities, such as optic pits, colobomas, and optic atrophy, or may occur as a result of elevated intraocular pressure [1].

Medical personnel should also be aware that anaesthesia, which includes nitrous oxide, is contraindicated in patients whose eyes are filled with gas. Nitrous oxide will quickly diffuse into the intraocular gas, causing expansion of the bubble in proportion to the concentration of nitrous being used. There are a number of cases of patients awakening from nitrous oxide anesthesia with permanent vision loss from arterial occlusion in an eye that contained a gas bubble [6–8]. Moreover, it should be noted that any gas fill of the eye has the same implications for nitrous oxide use.

Finally, it should be remembered that patients who are experiencing severe eye pain or elevated intraocular pressure from any cause may be subject to the oculocardiac reflex. Patients may develop nausea, vomiting, hypotension, bradycardia, and even cardiac arrest, all of which may resolve when the ocular problem is treated [9]. In the case presented, it is possible that the elevated eye pressure and pain may have contributed to her cardiac presentation. Timely treatment of the eye may have ameliorated the systemic problems.

## 4. Conclusion

When a patient with recent retinal surgery presents to the ED, it is important to determine if a gas bubble has been placed in the eye. In order to facilitate this, patients are given a bracelet to inform caregivers of the presence of intraocular gas—but this is not always present. The retinal surgeon should be contacted, especially if the patient needs treatment that may interfere with proper positioning. It should also be kept in mind that ocular—and even systemic—symptoms may be related to elevated pressure and should not be attributed to “normal” postoperative discomfort without an evaluation. Appropriate management of these patients will not only help ensure success of the retinal surgery; it can also avoid irreversible vision loss and may well help control their systemic medical problems.
